# Saikosaponin D attenuates inflammatory response and cell apoptosis of lipopolysaccharide‐induced lung epithelial cells

**DOI:** 10.1111/crj.13688

**Published:** 2023-08-24

**Authors:** Lijie Song, Guoyu Lu, Yanyan Tao

**Affiliations:** ^1^ Department of Emergency medical The First Affiliated Hospital of Bengbu Medical College Bengbu China

**Keywords:** acute lung injury, apoptosis, inflammation, Saikosaponin D, sepsis

## Abstract

**Background:**

Acute lung injury (ALI) is a prevalent complication of sepsis with high mortality rate. Saikosaponin D (SSD) is a triterpenoid saponin that has been reported to alleviate sepsis‐triggered renal injury in mice. Nonetheless, the therapeutic effect of SSD on sepsis‐evoked ALI is unclarified.

**Methods:**

Lipopolysaccharide (LPS) from *Escherichia coli* 055:B5 was utilized to stimulate lung epithelial cell line MLE‐12. A mouse model of sepsis was established. CCK‐8 assay was employed for determining cytotoxicity. ELISA was utilized for determining proinflammatory cytokine production. Flow cytometry and western blotting were implemented for evaluating cell apoptosis. Hematoxylin–eosin staining was conducted for histologic analysis of murine lung tissues.

**Results:**

SSD alleviated LPS‐triggered inflammation and cell apoptosis of MLE‐12 cells. SSD treatment ameliorated the pathological damages, inflammatory response, and cell apoptosis in the lungs of septic mice.

**Conclusion:**

SSD protects against sepsis‐triggered ALI by inhibiting inflammation and cell apoptosis in MLE‐12 cells and septic mouse mice.

## Graphical abstract

Lipopolysaccharide (LPS) induces inflammatory response and apoptosis of lung epithelial cells (MLE‐12). Saikosaponin D (SSD) treatment can reduce proinflammatory cytokine production and alleviate cell apoptosis in LPS‐stimulated MLE‐12 cells and animal models with acute lung injury.

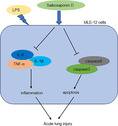

## 1 INTRODUCTION

Sepsis is defined as life‐threatening organ dysfunction caused by a dysregulated host response to infection.^1^ It is a main reason for death in patients with critical illness.^2^ Severe sepsis often results in multiple organ dysfunction syndrome.^3^ The lung is particularly vulnerable during sepsis and approximately 40% of acute lung injury (ALI) cases are attributed to sepsis.^4^ It has been indicated that the progression of sepsis‐triggered ALI is closely associated with pulmonary inflammation and cell apoptosis.^5^, ^6^ Elevation of inflammation and apoptosis in the lung leads to alveolar epithelial cell destruction, increased epithelial permeability, and influx of edematous fluid into the alveolar space.^6^ It has been indicated that proinflammatory cytokines, such as tumor necrosis factor (TNF)‐α and interleukin (IL)‐1β, are highly upregulated during sepsis‐induced ALI, contributing to vascular dysfunction and alterations in bronchomotor tone.^7^ Thus, inhibition of pulmonary inflammation and apoptosis may be a promising strategy for amelioration of sepsis‐triggered ALI.

Saikosaponin D (SSD) is a main bioactive component of the herbal medicine Radix bupleuri and possesses various pharmacological activities, such as anti‐inflammatory, anti‐cancer and metabolic regulation.^8^, ^9^, ^10^ Previous reports suggested that SSA, the epimer of SSD, alleviates lipopolysaccharide (LPS)‐evoked ALI in a mouse model.^11^ Wang et al. demonstrated that SSD improves mechanical ventilation‐triggered lung injury in rat models by alleviating inflammation, apoptosis and oxidative stress.^12^ Intriguingly, it has been illuminated that SSD ameliorates sepsis‐triggered renal cell apoptosis and inflammation in mice by repressing MMP9 expression via TCF7/FOSL1 pathway.^13^ Nonetheless, the therapeutic effect of SSD on ALI caused by sepsis is unclarified.

Herein, we aimed to explore the impact of SSD on sepsis‐evoked ALI. An LPS‐induced cell model and a mouse model of sepsis were established based on previous description. It was speculated that SSD might exert a protective role by suppressing lung inflammation and cell apoptosis. The results might help to develop new therapeutic ideas for ALI caused by sepsis.

## 2 MATERIALS AND METHODS

### 2.1 Preparation of SSD

SSD (purity >98%) was purchased from MedChemExpress (HY‐N0250, Shanghai, China) and dissolved in dimethyl sulfoxide (DMSO; Solarbio, Beijing, China).

### 2.2 Cell culture and treatment

Murine lung alveolar epithelial cell line MLE‐12 was obtained from WheLab (Shanghai, China) and incubated in DMEM/F12 (Gibco, Grand Island, NY, USA) containing 10% fetal bovine serum (FBS; Gibco) at 37°C in a humidified atmosphere with 5% CO_2_. To explore SSD impact on sepsis‐induced lung epithelial cell injury, MLE‐12 cells were incubated with 1 mg/mL LPS (L8880, Solarbio; *Escherichia coli* 055:B5) for 24 h with or without SSD (1, 2, and 4 μM).

### 2.3 Cell counting kit‐8 (CCK‐8) assay

MLE‐12 cells were inoculated into 96‐well plates (5 × 10^3^ cells/well) and incubated for 24 h at 37°C. Then, the cells were incubated with various doses of SSD (0–16 μM) for 24 h. Afterwards, 10 μL of CCK‐8 solution (Beyotime, Shanghai, China) was added followed by another 2‐h incubation. A microplate reader (Thermo Scientific, Waltham, MA, USA) was employed for measuring the 450‐nm absorbance. Cell viability was determined as a percentage of absorbance relative to the control. The experiment was conducted in triplicate.

### 2.4 Mice

Male C57BL/6 mice (8 weeks, 22–24 g) from Vital River (Beijing, China) were housed under specific pathogen‐free conditions with free access to food and water. The mice were allowed for 1‐week acclimation before experiments. All animal experiments were conducted following the NIH Guide for the Care and Use of Laboratory Animals and approved by the Ethics Committee of The First Affiliated Hospital of Bengbu Medical College. All efforts were made to minimize animals' sufferings.

### 2.5 Animal treatment and grouping

Thirty‐two mice were randomly grouped as follows (eight mice per group): (1) sham + control, (2) sham + SSD, (3) ALI + control, (4) ALI + SSD. A cecum ligation and perforation (CLP)‐induced mouse septic ALI model was established as previously reported.^14^ Briefly, mice were anesthetized by intraperitoneal injection of sodium pentobarbital (40 mg/kg). Then, a midline incision was made on the abdomen to expose cecum. The cecum was exteriorized and ligated at 1 cm from the cecal tip and perforated twice with a 20G needle 0.5 cm from the ligation site. Then the cecum was squeezed to extrude a small amount of feces and replaced in the abdominal cavity. The incision was sutured by layers. Mice in the sham group received the same procedures without cecum ligation or perforation. All mice were subcutaneously injected with normal saline (1 mL) immediately after the surgery for fluid resuscitation. After CLP induction, mice were intragastrically administrated with SSD (4 mg/kg) or control DMSO daily for five consecutive days. Five days after drug treatment, all mice were sacrificed under anesthesia. Bronchoalveolar lavage fluid (BALF) and lung tissues were harvested.

### 2.6 Lung wet/dry (W/D) ratio

The W/D ratio was measured for pulmonary edema evaluation as previously reported.^15^ Briefly, the lungs were weighed to measure the wet weight, and were then dried at 65°C for 72 h to get the dry weight.

### 2.7 Hematoxylin–eosin (H&E) staining

Fresh lung tissues were fixed in 4% paraformaldehyde, embedded in paraffin and cut into sections (5‐μm‐thick). Tissue sections were then deparaffined in xylene for 5 min, rehydrated in gradient alcohol and immersed in ddH_2_O for 2 min. After that, the sections were stained with hematoxylin (Solarbio) for 15 min, rinsed twice with tap water, and then stained with eosin for 2 min. After dehydrated, transparent and sealed with neutral resin (Sigma‐Aldrich), the stained lungs were observed using a microscope (Nikon, Tokyo, Japan).

### 2.8 Enzyme‐linked immunosorbent assay (ELISA)

The concentrations of proinflammatory cytokines in cell supernatant and BALF samples were determined using mouse ELISA kits (Abcam, Shanghai, China): IL‐6 (ab222503), IL‐1β (ab197742), and TNF‐α (ab208348), according to the manufacturer's protocols.

### 2.9 Flow cytometry

Flow cytometry was used for cell apoptosis analysis using Annexin V‐FITC/PI Apoptosis Detection Kit (Beyotime). MLE‐12 cells were washed with PBS, centrifuged at 1000*g* for 5 min and resuspended in 195‐μL 1× binding buffer. Then the cells were treated with 5‐μL Annexin V‐FITC and 10‐μL propidium iodide (PI) solution for 10 min at room temperature without light. Cell apoptosis was examined using a flow cytometer (BD Biosciences, Franklin Lakes, NJ, USA). Cells were gated according to forward scatter/side scatter (FSC/SSC) parameters and flow cytometry analysis was performed using FlowJo software. Quadrants 2 (Q2) and Q3 were used for apoptosis analysis.

### 2.10 Western blotting

Proteins were extracted from MLE‐12 cells or murine lung tissues using RIPA lysis buffer (Solarbio). A bicinchoninic acid assay kit (Solarbio) was employed for protein concentration determination. Protein samples (20 μg) were dissolved by 10% SDS‐PAGE, blotted onto polyvinylidene fluoride membranes (Beyotime) and blocked with 5% nonfat milk. The membranes were incubated at 4°C overnight with primary antibodies against: caspase3 (ab184787, 1:2000, Abcam), cleaved‐caspase3 (ab214430, 1:5000, Abcam), caspase8 (ab227430, 1:500, Abcam), cleaved‐caspase8 (#8592, 1:1000, Cell Signaling, Shanghai, China) and GAPDH (ab22555, 1:1000, Abcam), and then incubated with the secondary antibody (ab97080, 1:5000, Abcam) for 1 h. Blot visualization was achieved using an ECL detection kit (Solarbio) and ImageJ software (NIH, Bethesda, MD, USA) was used for protein level quantification.

### 2.11 Statistical analysis

Data are expressed as mean ± standard deviation. Student's *t*‐test or one‐way ANOVA with Tukey's post hoc analysis were used for difference comparison among groups. Each experiment was repeated at least 3 times. Statistical analysis was performed using SPSS 25.0 software (IBM, Armonk, NY, USA), and *p* ˂ 0.05 indicated statistical significance.

## 3 RESULTS

### 3.1 Cytotoxicity of SSD to MLE‐12 cells

**FIGURE 1:**
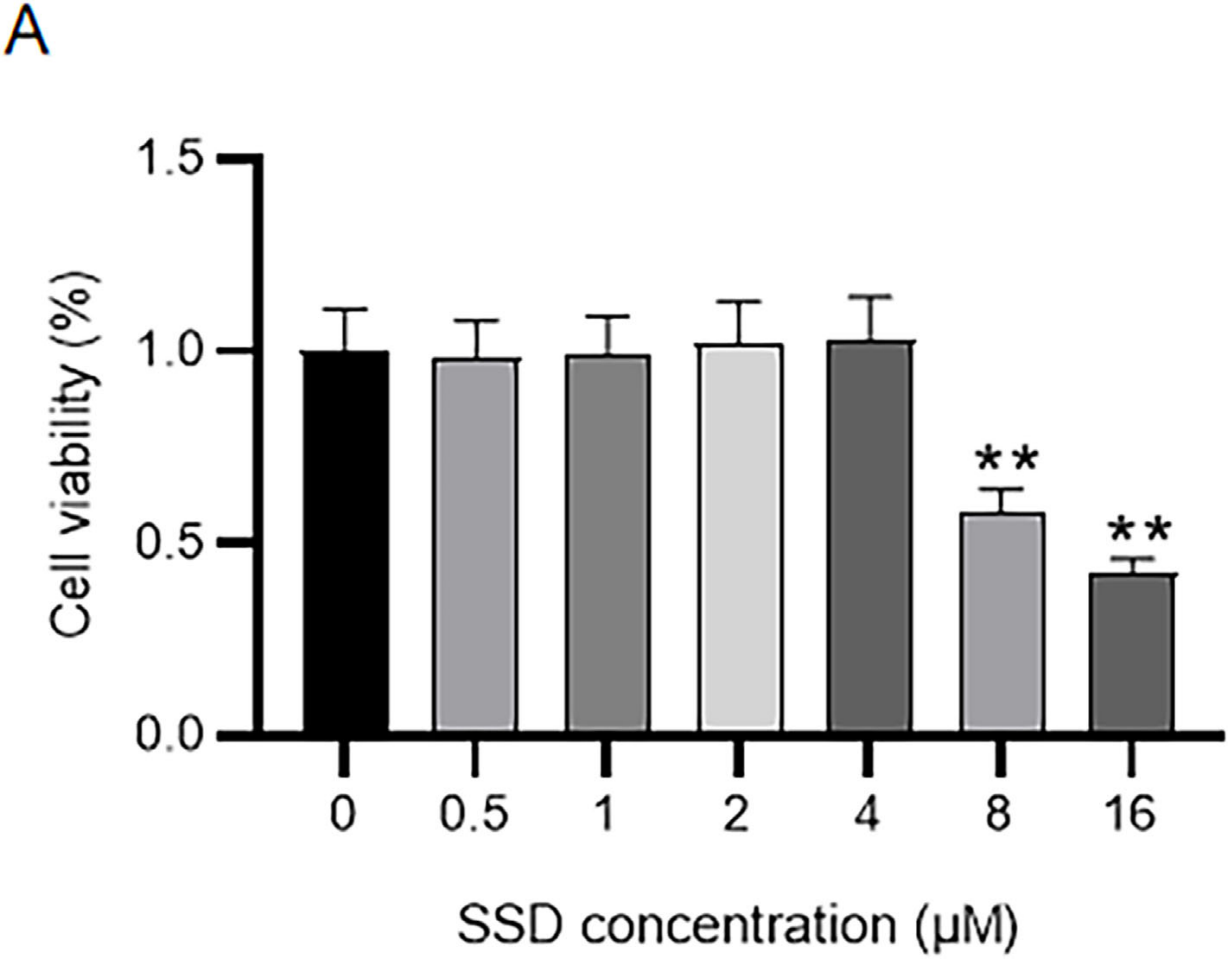
SSD effect on MLE‐12 cell viability. (A) CCK‐8 assay for examining the cytotoxicity of SSD to MLE‐12 cells. *n* = 3. Data were analyzed by Student's *t*‐test. ^**^ *p* < 0.01 versus 0‐μM SSD group.

We first examined the cytotoxicity of SSD to MLE‐12 cells. MLE‐12 cells were exposed to various concentrations of SSD for 24 h. As displayed in Figure 1A, SSD at concentrations up to 4 μM was non‐cytotoxic to MLE‐12 cells. SSD at doses of 8 or 16 μM significantly reduced MLE‐12 cell viability. Thus, 1‐, 2‐, and 4‐μM SSD were used for subsequent experiments.

### 3.2 SSD alleviates LPS‐triggered inflammation and apoptosis of MLE‐12 cells in vitro

**FIGURE 2:**
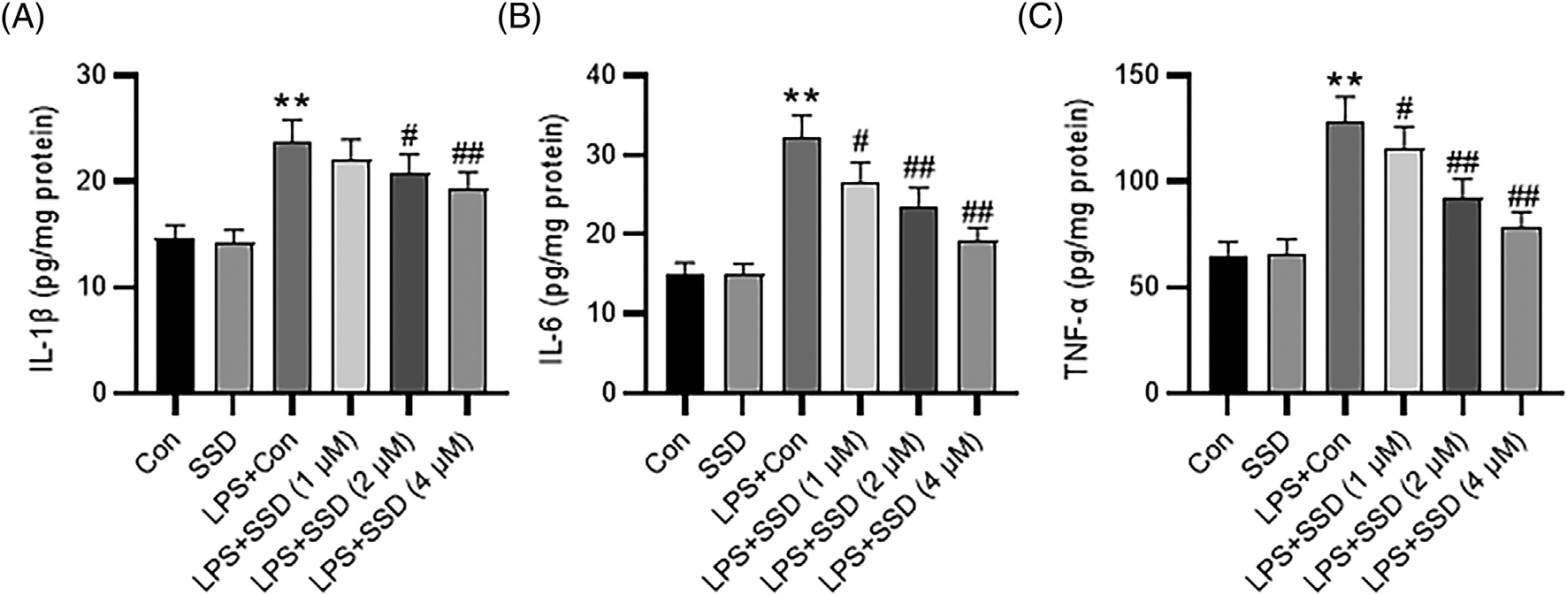
SSD alleviates LPS‐triggered inflammation in MLE‐12 cells. (A–C) ELISA for determining the concentrations of IL‐6, IL‐1β, and TNF‐α in MLE‐12 cells with indicated treatments. *n* = 3. Data were analyzed by one‐way ANOVA. ^**^ *p* < 0.01 versus the control group; ^#^ *p* < 0.05, ^##^ *p* < 0.01 versus LPS + Con group.

**FIGURE 3:**
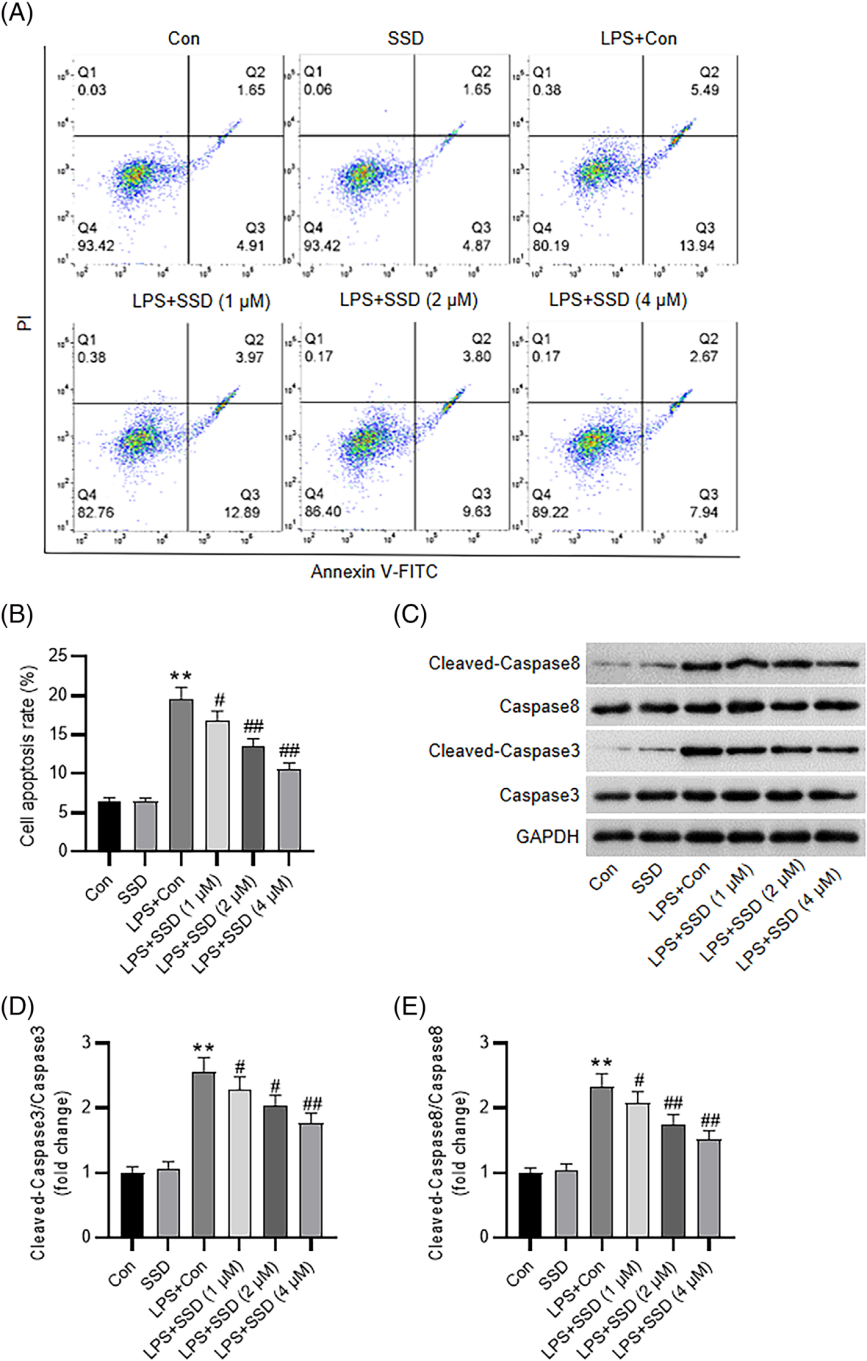
SSD attenuates LPS‐evoked MLE‐12 cell apoptosis. (A,B) Flow cytometry for evaluating MLE‐12 cell apoptosis. (C–E) Western blotting for measuring levels of apoptosis‐related proteins in indicated MLE‐12 cells. *n* = 3. Data were analyzed by one‐way ANOVA. ^**^ *p* < 0.01 versus the control group; ^#^ *p* < 0.05, ^##^ *p* < 0.01 versus LPS + Con group.

To explore SSD impact on lung epithelial cell injury, MLE‐12 cells were stimulated with LPS for 24 h with or without SSD treatment (1, 2, and 4 μM). ELISA revealed that LPS markedly elevated the secretion of proinflammatory cytokines in MLE‐12 cells, including IL‐6, IL‐1β, and TNF‐α. Nevertheless, the effects were abated by SSD treatment in a dose‐dependent manner (Figure 2A–C), indicating that SSD attenuated LPS‐triggered inflammation. Moreover, SSD markedly ameliorated LPS‐evoked MLE‐12 cell apoptosis, as demonstrated by flow cytometry (Figure 3A,B). Consistently, western blotting depicted that LPS stimulation facilitated the cleavage of pro‐apoptotic proteins caspase3 and caspase8 in MLE‐12 cells, while SSD dose‐dependently counteracted the above effects (Figure 3C–E). Collectively, these data suggested that SSD could improve LPS‐triggered inflammation and apoptosis of MLE‐12 cells.

### 3.3 SSD ameliorates CLP‐induced ALI in mice

**FIGURE 4:**
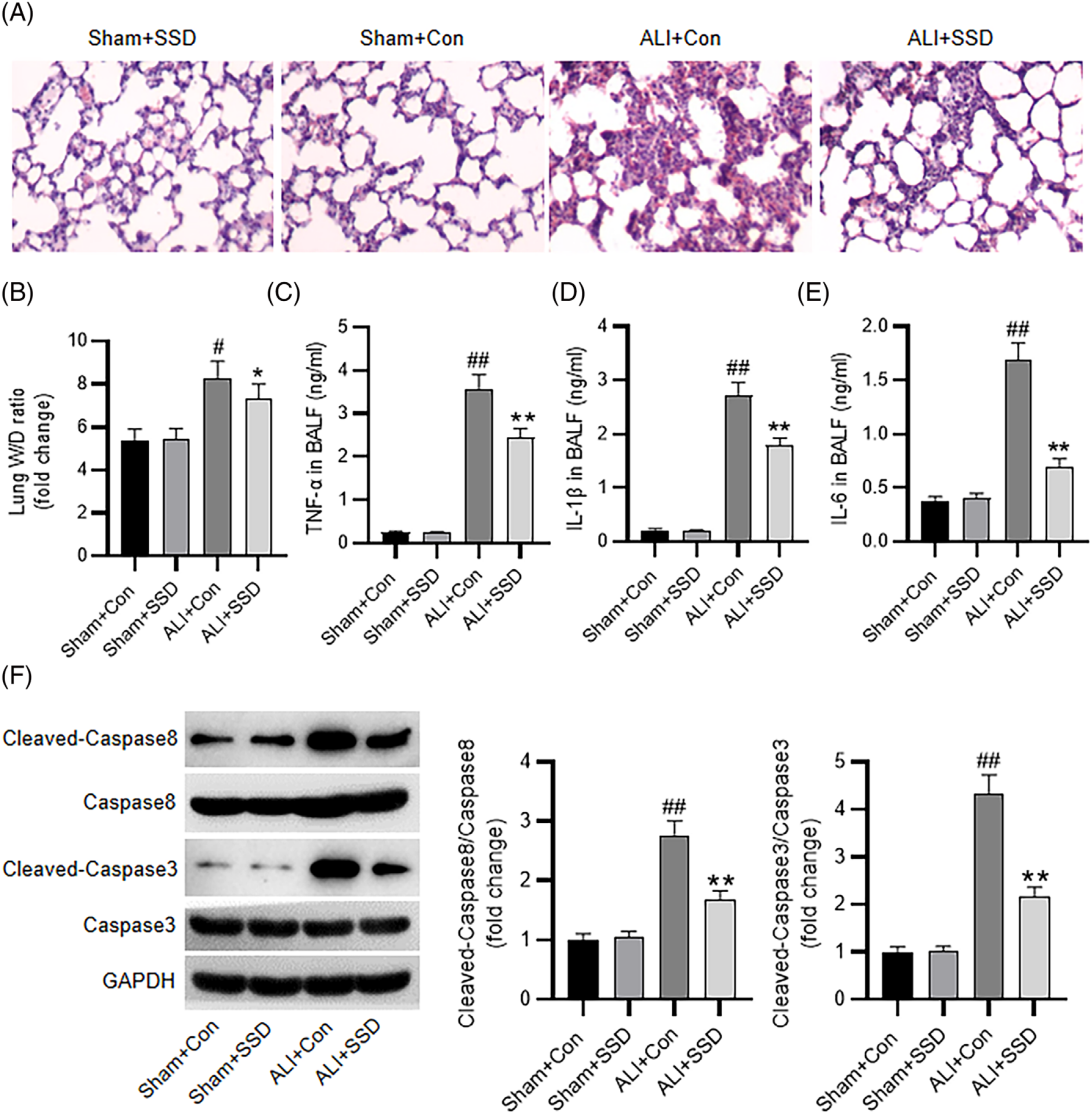
SSD ameliorates CLP‐induced ALI in mice. (A) Representative images of H&E staining for histological analysis of murine lung tissues. (B) Measurement of lung W/D ratio. (C–E) ELISA for determining concentrations of proinflammatory cytokines in BALF. (F) Western blotting for examining expression of apoptosis‐related proteins in the lung tissues. *n* = 4. Data were analyzed by one‐way ANOVA. ^#^ *p* < 0.05, ^##^ *p* < 0.01 versus the sham + Con group; *p < 0.05, ^**^ *p* < 0.01 versus ALI + Con group.

To further identify SSD effect on sepsis‐associated ALI, a mouse ALI model was established via CLP with or without administration of SSD. H&E staining was implemented for histological analysis of murine lung specimens. In comparison to those of the sham‐operated mice, the lung tissues of septic mice were severely damaged, as indicated by alveolar wall thickening, inflammatory cell infiltration, edema and hemorrhage. However, SSD treatment markedly improved the above pathological changes in ALI mice (Figure 4A). W/D ratio of the lung was measured for the evaluation of pulmonary edema. Consistent with the histologic analysis, the results showed that SSD markedly reduced W/D ratio of the lung in CLP‐triggered ALI mice (Figure 4B). Moreover, SSD administration decreased the high levels of proinflammatory cytokines (TNF‐α, IL‐6, IL‐1β) in BALF of CLP‐induced ALI mice, as suggested by ELISA (Figure 4C–E). Additionally, we measured levels of apoptosis‐related proteins in the lungs. As indicated by western blotting, levels of cleaved‐caspase3 and ‐caspase8 in CLP‐evoked mice were prominently higher than those in sham‐operated mice, whereas SSD administration decreased the high protein levels (Figure 4F), suggesting that SSD alleviated cell apoptosis in murine lung tissues. The above data indicated that SSD could ameliorate CLP‐triggered ALI in mouse models.

## 4 DISCUSSION

Sepsis is a critical global health issue, and ALI is a common complication of sepsis with high death rate.^16^ SSD is a triterpenoid saponin that has been reported to alleviate cell apoptosis and renal inflammation in septic mice.^13^ Herein, we investigated the effects of SSD on sepsis‐evoked ALI via an LPS‐stimulated MLE‐12 cell model and a mouse sepsis model.

LPS is a dominant outer membrane component of gram‐negative bacteria and acts as a major pathogenic factor of sepsis.^17^ It is well established that LPS induces inflammation cascade, leading to apoptosis of lung epithelial cells.^18^ Consistently, our results revealed that LPS prominently promoted inflammation in MLE‐12 cells, as indicated by proinflammatory cytokine upregulation. High serum levels of TNF‐α and IL‐6 have been reported in septic mice with ALI.^19^ However, the effects were abated by SSD treatment, suggesting the anti‐inflammatory effect of SSD, which is in line with previous reports.^13^, ^20^ Additionally, our results demonstrated that SSD at a concentration of 4 μM was non‐cytotoxic to lung epithelial cells in vitro.

Caspases are the core components involved in apoptosis mechanism.^21^ Caspase8 is an initiator caspase widely expressed in most human and rodent cells.^22^ Pro‐caspase8 is auto‐cleaved to produce active caspase8, which subsequently initiates the execution phase of apoptosis. Moreover, active caspase8 cleaves caspase3 to promote proteolytic degradation of intracellular proteins.^23^ In the present study, our results demonstrated that SSD reduced the cell apoptosis rate and significantly repressed the cleavage of caspase3 and caspase8 in MLE‐12 cells under LPS stimulation. Previous evidence has illustrated that SSD attenuates renal cell apoptosis in septic mice.^13^ SSD suppresses acinar cell apoptosis by inactivating MAPK signaling pathway, thereby ameliorating pancreatic injury.^24^ These reports support our findings in this study.

Several reports have indicated that SSD protects against lung injury in animal models and exerts an antitumor role in lung cancer.^12^, ^25^ Consistent with the above in vitro results, our animal experiments elucidated the protection effect of SSD on sepsis‐triggered ALI. It was depicted that SSD administration alleviated inflammatory cell infiltration, edema as well as cell apoptosis in the lungs of septic mice.

In conclusion, this study revealed that SSD could alleviate LPS‐triggered inflammation and apoptosis of MLE‐12 cells and ameliorate sepsis‐evoked pathological damages in the lungs of mice. Our findings might help to develop a new therapeutic agent for ALI caused by sepsis. In addition, further investigations are required to explore the potential mechanisms underlying the protection effect of SSD in sepsis‐induced ALI.

## AUTHOR CONTRIBUTIONS

Lijie Song were the main designers of this study. Lijie Song, Guoyu Lu, and Yanyan Tao performed the experiments and analyzed the data. Lijie Song and Yanyan Tao drafted the manuscript. All authors read and approved the final manuscript.

## CONFLICT OF INTEREST STATEMENT

The authors declare no competing interests.

## ETHICS STATEMENT

All animal experiments were conducted following the NIH Guide for the Care and Use of Laboratory Animals and approved by the Ethics Committee of the First Affiliated Hospital of Bengbu Medical College.

## Data Availability

The data underlying this article will be shared on reasonable request to the corresponding author.
